# A case study of using natural language processing to extract consumer insights from tweets in American cities for public health crises

**DOI:** 10.1186/s12889-023-15882-7

**Published:** 2023-05-24

**Authors:** Ye Wang, Erin Willis, Vijaya K. Yeruva, Duy Ho, Yugyung Lee

**Affiliations:** 1 Department of Communication and Journalism, University of Missouri-Kansas City, 202 Haag Hall, 5120 Rockhill Road, 816-235-2735, Kansas City, MO 64110 USA; 2 Department of Advertising, Public Relations & Media Design, University of Colorado Boulder, 478 UCB, 1511 University Avenue, Boulder, CO 80309-0200 USA; 3 Division of Computing, Analytics, and Mathematics, University of Missouri-Kansas City, 801 E51st St, Kansas City, MO 64110 USA

**Keywords:** Health communication, Vaccine hesitancy, Natural language processing, Twitter, Public health crisis, COVID 19

## Abstract

**Background:**

The COVID-19 pandemic was a “wake up” call for public health agencies. Often, these agencies are ill-prepared to communicate with target audiences clearly and effectively for community-level activations and safety operations. The obstacle is a lack of data-driven approaches to obtaining insights from local community stakeholders. Thus, this study suggests a focus on listening at local levels given the abundance of geo-marked data and presents a methodological solution to extracting consumer insights from unstructured text data for health communication.

**Methods:**

This study demonstrates how to combine human and Natural Language Processing (NLP) machine analyses to reliably extract meaningful consumer insights from tweets about COVID and the vaccine. This case study employed Latent Dirichlet Allocation (LDA) topic modeling, Bidirectional Encoder Representations from Transformers (BERT) emotion analysis, and human textual analysis and examined 180,128 tweets scraped by Twitter Application Programming Interface’s (API) keyword function from January 2020 to June 2021. The samples came from four medium-sized American cities with larger populations of people of color.

**Results:**

The NLP method discovered four topic trends: “COVID Vaccines,” “Politics,” “Mitigation Measures,” and “Community/Local Issues,” and emotion changes over time. The human textual analysis profiled the discussions in the selected four markets to add some depth to our understanding of the uniqueness of the different challenges experienced.

**Conclusions:**

This study ultimately demonstrates that our method used here could efficiently reduce a large amount of community feedback (e.g., tweets, social media data) by NLP and ensure contextualization and richness with human interpretation. Recommendations on communicating vaccination are offered based on the findings: (1) the strategic objective should be empowering the public; (2) the message should have local relevance; and, (3) communication needs to be timely.

**Supplementary Information:**

The online version contains supplementary material available at 10.1186/s12889-023-15882-7.

## Background

Vaccine hesitancy is a recurring theme challenging the world’s public health [1]; often, the solution lies in health communication [2] and subsequent policymaking [3]. “Effective communication strategies are important to engage policymakers and communities in prevention and control efforts, and to increase buy-in and confidence” [4]. In a letter to the editor of *Psychological Medicine*, Barello et al. [2] called for more research to “look inside the ‘black box’ of vaccine hesitancy” (p. 701). This call was timely given the urgence of vaccination in 2020 after COVID-19 emerged. However, it was also concerning that months after global efforts to vaccinate the world’s population, there was not a good understanding of consumer insights about those who chose to be vaccinated and those who refused [2]. This contrast suggested that many vaccination campaigns during the pandemic were running on assumptions rather than data-driven approaches to consumer insights.

### Related studies on the sources of vaccine hesitancy

To understand vaccine hesitancy, studies have been done at national and global levels to analyze its sources in Australia, United Kingdom, South Korea, etc. Due to the large amount of text data, these studies applied Natural Language Processing (NLP) to algorithmically analyze text data. Shim, Ryu, Lee, et al. [5] applied Latent Dirichlet Allocation (LDA) topic modeling to 3,509 tweets from Korea and discovered that COVID-19 vaccine hesitancy was a popular topic of discussion. People discussed the safety of the vaccine and degree of symptoms experienced [5]. Kwok, Vadde, and Wang [6] found from 31,100 tweets that Australian users largely supported infection control measures and refuted misinformation, but some were influenced by conspiracy theories. Cotfas, Delcea, Roxin, et al. [7] discovered a largely neutral stance of people in the United Kingdom toward lockdown measures in November 2020 based on over 2 million tweets. Lyn, Le Han, and Luli [8] analyzed 1,499,421 tweets and revealed global trends of opinions on vaccination.

In comparison to the NLP methods, the strengths of surveys, content analyses, and textual analyses are to add context and depth to the understanding of vaccine hesitancy. A nationwide survey of 1,005 Italians conducted between November 27 and December 3, 2020, showed that confidence in the safety of the vaccine played a major role in affecting vaccine hesitancy, and collective responsibility had only marginal importance. The researchers argued that vaccination campaigns should aim at increasing individual’s trust in the effectiveness and safety of vaccines; trust is also key to discrediting conspiracy theories [2]. Conducting content analysis on a national sample of tweets, Griffith, Marani, and Monkman [1] identified major themes of vaccine hesitancy in Canada, including political skepticism, lack of knowledge, distrust toward authorities and institutions, distrust in the legal system, and the legacy of harm caused by health care institutions on people of color. Such contextual information suggests that no one can take public trust for granted, especially not health authorities [9].

### Public trust and one-way communication

Trust is monumental to vaccination campaigns targeting African Americans yet challenging to create among this population. As pointed out by Salmon, Opel, Dudley, et al. [10] the pandemic has affected African American communities more so than other communities, compounded by the memory of the historical and cultural experience with experimental medical research on people of color. Additionally, trust may be put on shaky ground simply because health institutions are perceived as powers that individuals face up to [9]. This feeling may sometimes resonate with the suppression that African Americans have experienced historically. Additionally, the public is increasingly aware of the uncertainty of scientific knowledge and the lack of scientific consensus [11, 12]. Thus, depicting the vaccine refusers as uneducated may misguide communication efforts against disinformation [9]. Moreover, this one-way communication of being told “what to do” and “what to believe” may unintentionally fuel conspiracy theories: health agents are just part of the suppressive institution. Thus, vaccination campaigns were urged to discredit conspiracy theories by emphasizing individuals’ trust in the effectiveness and safety of vaccines [2].

Enhanced trust is a result of inclusiveness of all stakeholders, mutual understanding, and the recognition of different perspectives [13]. In the ecology of public health [14], an individual’s health decision-making is a result of multiple levels of influence; and informational environments play an important role. Undeniably, digital technologies, for example, Twitter, Instagram, blogs and websites, have posed challenges to the “central position” of public health agencies in informing the public. In a large ecological system of public health, individuals’ health decision-making is influenced by interactions and exchanges occurring in the entire informational and social environments, including mass media, health organizations, search engines, social media, etc. [14, 15]. For better or for worse, information technologies like social media and algorithmic curation have weaken the control of the traditional health information centers (for example, the Centers for Disease Control and Prevention) due to misinformation [15], or the amplification of the voices of small, individual stakeholders. The “digital destruction” interrupts and degrades the command-and-control channel between crisis management agencies and the public. Facing a highly competitive marketplace of information, public health crisis management must not solely count on the command-and-control approach to health communication. Rather, understanding and exploiting two-way communication is key to future success of health communication.

### Two-way communication

Two-way communication closes the feedback loop of listening to stakeholders and recognizing their needs. Kim et al. [16] described two-way communication on social media as processes of listening to, targeting, and reaching diverse audiences. Social media are “sandboxes” for people to form narratives about health [17]. Users share their personal experiences and feelings about health [18]. Thus, online social networks become sources of psychological support and social persuasion [19]. High interactivity on social media can potentially improve credibility perception of public health agencies during outbreaks [20]. The benefit of two-way communication motivates more and more public health agencies to adopt social media to engage with the public [16].

In recent years, technology advancement in NLP has made it possible to “listen” to many stakeholders on social media at national and global levels, to have a firmer grasp on the complex ecological systems of public health. This increase of analytical capacity has energized infodemiology studies that assess health-related topics using web-based data to perform a surveillance function for public health agencies [20]. During the COVID-19 pandemic, AI technologies demonstrated potential thanks to the copious amounts of data available on social media. Amid the proliferation of infodemiology studies, this study urges a focus on listening at local levels, for example, cities, given the abundance of geo-marked data, and the importance of community-level safety operations to manage public health crises. Unlike the surveillance purpose of infodemiology, listening to local communities aims at enhancing two-way communication. Local communities can be viewed as multiple stakeholder systems. A community has geographical boundaries, and the community members are connected via social relationships [21]. By obtaining insights into multiple stakeholders, communication strategies can be identified to guide local public health campaigns for positive health outcomes within the community.

### Purposes

The goal of this study is to demonstrate how to combine human and NLP machine analyses to reliably extract valid consumer insights from social media content. The strength of machine analyses is quick reduction of large amounts of text data into semantic or emotional categories. The strength of human analyses is contextualization of consumer insights. This study demonstrates a process of combining the strengths of both approaches to analyze large amounts of social media content. The research question that guides this study is:

- RQ: How do we combine machine and human analysis to reliably extract valid consumer insights from social media content?

The method section will present a data process flow that integrates NLP and human textual analyses. Justifications for the design of the process are offered to demonstrate how machine and human analyses can serve the greater objective of understanding the perspectives of multiple stakeholders for community health.

## Methods

### The aim

The goal of this case study is to understand consumer insights of COVID-19 vaccines in medium-sized cities that have higher numbers of people of color, with a focus on Kansas City, Missouri.

### The setting of the study

At the beginning of August, the COVID-19 vaccination (two shots) rate in Missouri was 41.5%. However, due to the Delta variant, Kansas City was identified as having a lower vaccination rate and a rising number of new cases. Accordingly, local healthcare leaders began to pursue a more targeted approach to vaccination campaigns and address health disparities that impeded Kansas City’s vaccination rate progress. In particular, a few zip code areas and surrounding communities on the east side of Kansas City (64106, 64109, 64127, 64128, 64129, and 64130) were prioritized as the focal point of the Our Healthy KC Eastside Campaign (OHEKC), an initiative funded by Jackson County (see Table 1). These areas have more African Americans [22] (U.S. Census, 2019) and are experiencing disproportionate health outcomes related to COVID-19 due to low vaccination rates [23]. For example, a report by KCUR [24] noted that as of May 2021, the population of zip code 64130, 88% African American, had a 15% partial vaccination (one-shot) rate.

**Table 1:** Demographics of the targeted zip codes and their vaccination rates in May 2021

Zip code in KC	Percentage of African Americans	Partially vaccinated (at least one dose)		
		White	Black or African American	Hispanic or latino
64106	44%	22%	6%	17%
64109	48%	23%	18%	15%
64127	51%	11%	15%	9%
64128	81%	9%	14%	19%
64129	45%	12%	13%	9%
64130	88%	13%	15%	28%

This study was conducted prior to OHEKC, to obtain consumer insight and inform the communication of this campaign. Three additional cities were included in the data collection for two reasons. First, the breadth of the data collection could be increased by sampling from similar markets. These selected cities are like Kansas City in terms of city size and the proportion of the minority population. NLP models often perform better on a larger dataset. Second, it also gives an opportunity to demonstrate the strengths of human analysis in interpreting nuances.

**Fig. 1:**
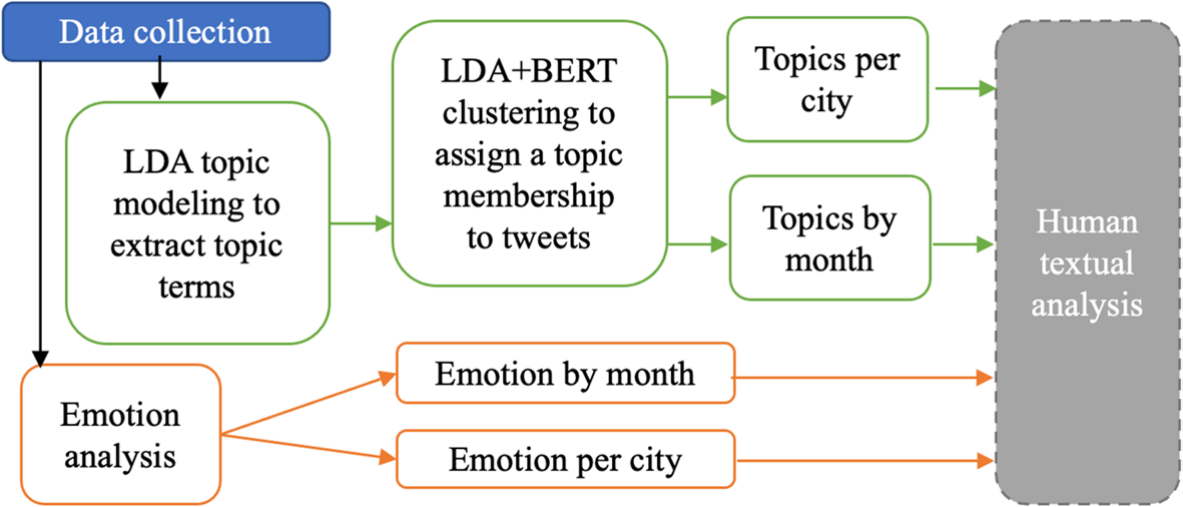
The workflow of the data analysis. (LDA stands for Latent Dirichlet Allocation. BERT stands for Bidirectional Encoder Representations from Transformers)

Figure 1 shows the pipeline of the data analysis. Due to the large number of tweets, NLP was used to perform initial data reduction and provide analytics on topics and emotion to inform human textual analysis. The human textual analysis offered contextualized interpretation of consumer insights in these four cities.

### Data collection

**Fig. 2:**
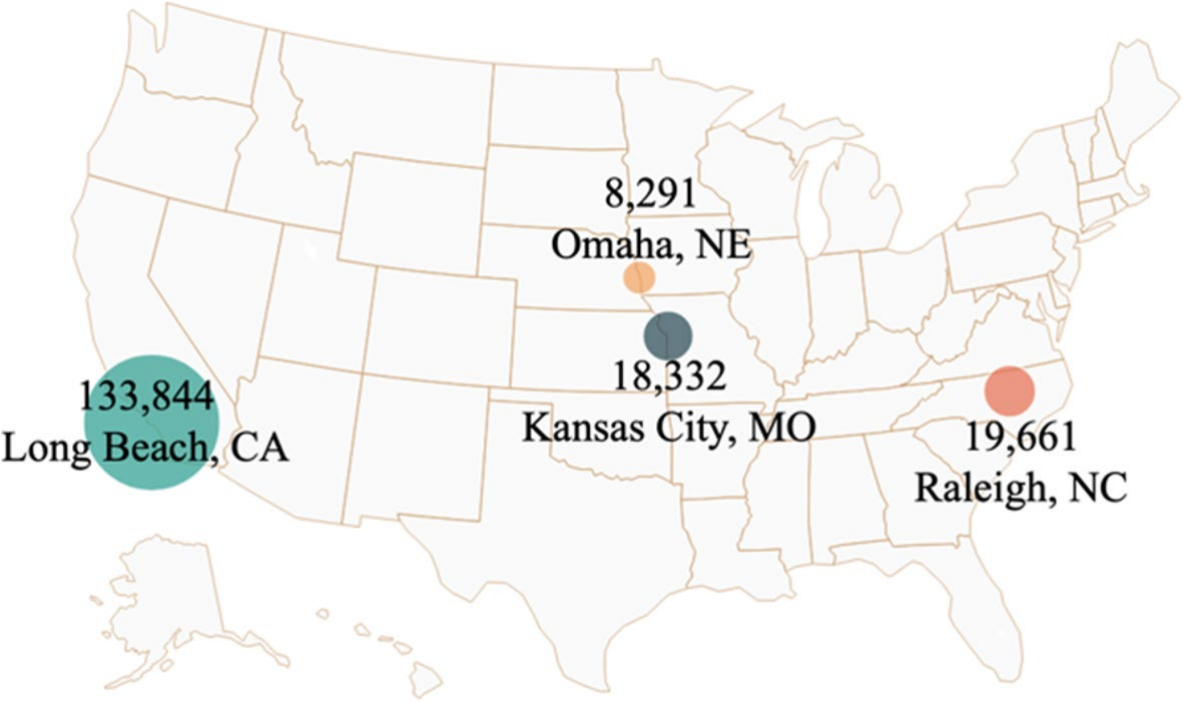
Geographic distribution of the sampled tweets

The data collection was collected from January 2020 to June 2021, covering the beginning, the peaks, and the subsiding spread of the coronavirus (see Fig. 2). We collected data from Twitter since it is widely used in the US [25]. According to recent statistics, 21% of US adults are Twitter users [26]. Representation of Black and Hispanic users is similar or slightly higher than the general population [27]. We used Twitter Application Programming Interface (API) and collected tweets from four cities in the US during the period between January 2020 and June 2021: 133,844 from Long Beach, California, 8,291 from Omaha, Nebraska, 18,332 from Kansas City, and 19,661 from Raleigh, North Carolina (see Fig. 2). To use Twitter API’s geo-location function, the research team filed an application to Twitter for review and was approved for a Twitter’s Researcher Account that allows geo-based scrapping. Our search criteria included: “COVID” or “coronavirus” in the tweet and geo-location from the United States. Additionally, we filtered only English tweets 20 miles around the point of interest (latitude and longitude of the cities).

### Data preprocessing

Initially, we converted the tweets to all lower-case characters, and then removed special characters and URLs from the tweet. As a result, the corpus contained only words that had substantial meaning. For the same reason, tweets that had single or double words were removed together with “stop words,” for example, “the,” “no,” or "I did.” After preprocessing, there was 0.1% of data loss from the raw tweets.

### Topic modeling

LDA topic modeling was used to identify latent semantic spaces, or in other words, underlying trends in the text data. LDA is a generative probabilistic model based on words, topics, and documents [28]. It computes per-topic word distribution as a representation of topics (latent semantic trends), and per-document topic distribution as a representation of document semantics in relation to the discovered “topics.” For technical details, please see the Appendix.

Generally, a researcher will choose the number of topics that generates a high coherence score and produces interpretable topics. The number of topics is a hyperparameter chosen by human experts to initialize the model. The final topic number is selected based on coherence score analysis and human evaluation of interpretability. The coherence score analysis compares the coherence scores of topic models given different numbers of topics. It uses normalized pointwise mutual information (NPMI) and cosine similarity to measure topic coherence [29]. NPMI is a normalized measure of co-occurrence probabilities of words. The human evaluation will qualitatively measure whether the extracted topic key words are meaningful and coherent.

Once the number of topics is selected, pyLDA visualization can be used to assist in naming each topic. pyLDA visualizes semantic distance between topics, relevance of top topic words to each topic, and the size of each topic [30]. A human researcher will examine the most relevant and most frequent key words in pyLDA to form initial interpretation/names of each topic. To finalize topic names, the human researcher will examine original tweets to ensure the consistency between a topic name and topic content. During this process, topics could be dropped due to lack of interpretability. Additionally, if the topic size is too small to represent a recurring theme, it could be dropped from further interpretation.

### Clustering tweets

The LDA topic model provides a probability distribution of a tweet across all topics. However, it does not assign a tweet to a topic category. Coding all tweets into topic categories is extremely time-consuming and labor-intensive for human coders. Instead, this study applied LDA + BERT (Bidirectional Encoder Representations from Transformers) clustering [31] to automatically code documents into a topic category. K-Mean clustering is a dimension reduction technique that assigns a document to one of the k clusters. The motivation of mixing LDA document vector and BERT sentence vector is to address the inherent weakness of the bag-of-words (BoW) approach in statistical modeling (e.g., LDA topic modeling). BoW ignores word dependency, which cannot be addressed by techniques such as TF-IDF or additional feature engineering.

The strength of LDA topic models is to utilize global statistics of the corpus to identify latent semantic spaces (i.e., topics). However, the BoW approach ignores the sequential dependency that may impact the meaning of a word in a local context (i.e., a sentence). For example, a LDA topic model can capture the most probable words, for example, “covid,” “get,” “vaccine.” and “like,” of a latent semantic space. However, it does not provide any information on whether “get” means “understand” in “I got what you mean” or “receive” in “I got the vaccine.” As clustering is performed on each document, local understanding is important. Word embeddings learned by deep learning models such as Word2Vec and BERT utilize local contexts. They can introduce “local semantics” given a sequence/sentence. They can be used to balance out the weakness of document vectors computed by LDA topic models. This study chose pre-trained BERT embedding since it is trained on a large corpus and represents a “general” language aptitude that can disambiguate polysemy at the sentence level.

**Fig. 3:**
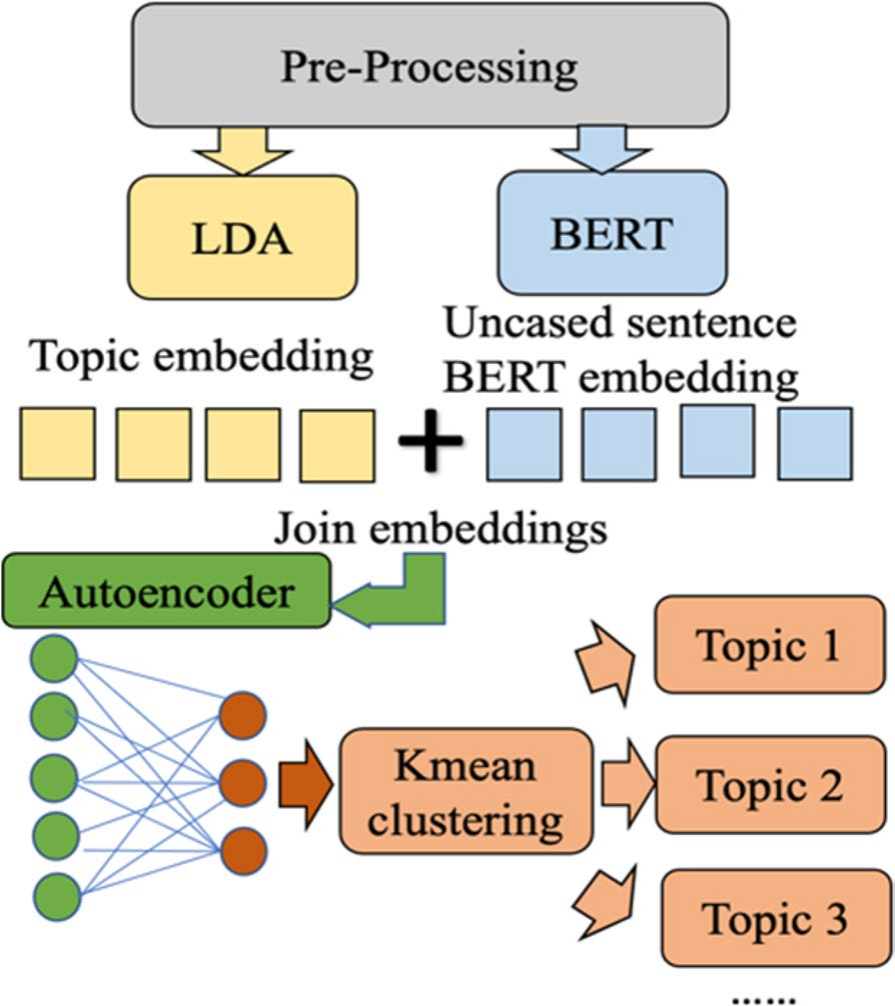
The LDA + BERT clustering model. (LDA stands for Latent Dirichlet Allocation. BERT stands for Bidirectional Encoder Representations from Transformers)

The specific steps of LDA + BERT clustering are shown in Fig. 3. First, the uncased pre-trained sentence BERT from the Hugging Face library [32] was used to generate a contextual representation of each tweet. Then, the sentence BERT embedding [33], concatenated with LDA sentence vectors were entered into a simple autoencoder with a dense layer. Next, each tweet was encoded into a latent vector space representation. Finally, K-Means clustering was applied to the latent representation and exclusively put a tweet into a topic category. We used the same number of topics k in LDA to do K-Mean clustering. Figure 3 shows the model architecture.

### Emotion analysis

The purpose of the emotion analysis is to give the human analyst a sense of the sentiment of the discussions. Emotion Analysis is an extension of sentiment analysis [34]. Emotion recognition helps us dig deeper into users' opinions on products, services, and benefits and understand conversations more precisely using deep learning models [35]. Regarding BERT's implementation, we have used the pre-trained BERT model from the Hugging Face library. This model has different text classification tasks, including sentiment, toxicity, and emotion. The emotion task has four labels "joy," "optimism," "sadness," "anger." To label the Twitter data, we have parsed each tweet according to the model. The output from the model is the single label from the list of emotions.

### Textual analysis

The purpose of the human textual analysis, informed by the topics and emotions assigned by the NLP models, is to offer a refined interpretation for each city. Berger [36] describes textual analysis as a way for researchers to gather information about how others make sense of the world. The textual analysis allowed for a local perspective to emerge from the tweets above general quantification. A human expert (one of the authors) conducted a textual analysis of 4,000 randomly selected tweets from January 1, 2020, through June 30, 2021. Each tweet had a date and a city of publishing, a topic membership assigned by the LDA + BERT model, and an emotion category assigned by the BERT emotion model. Tweets were read and analyzed longitudinally.

Tweets were examined several times: themes emerged during initial readings, and additional readings were done to explore those themes [37]. Miles and Huberman [38] instruct researchers to isolate themes “(a) that happen a number of times and (b) that consistently happen in a specific way” (p. 215). Based on location information, the authors summarized the discussion in the four cities regarding COVID-19 and its vaccination.

## Results

This study examined consumer insights of COVID-19 vaccines from Twitter across four cities with higher numbers of African Americans for the purpose of understanding vaccine hesitancy and informing public health campaigns.

### Topic modeling

**Fig. 4:**
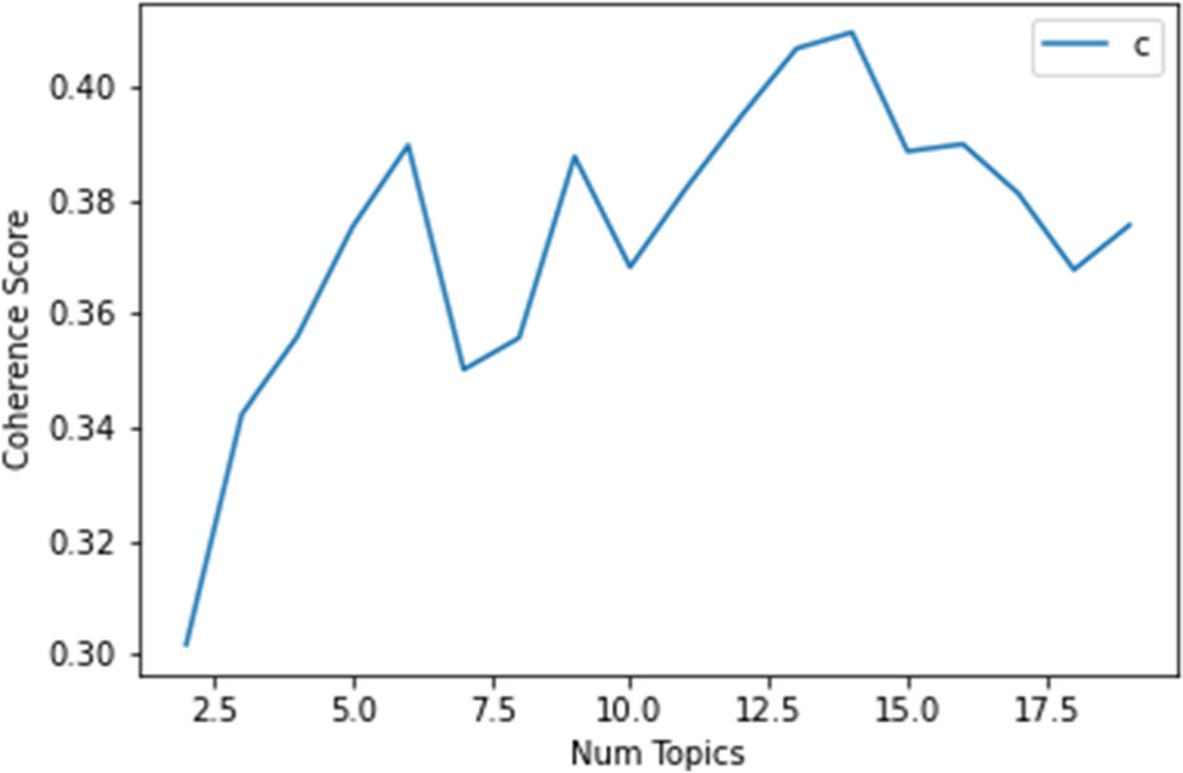
Coherence scores of the numbers of topics

**Fig. 5:**
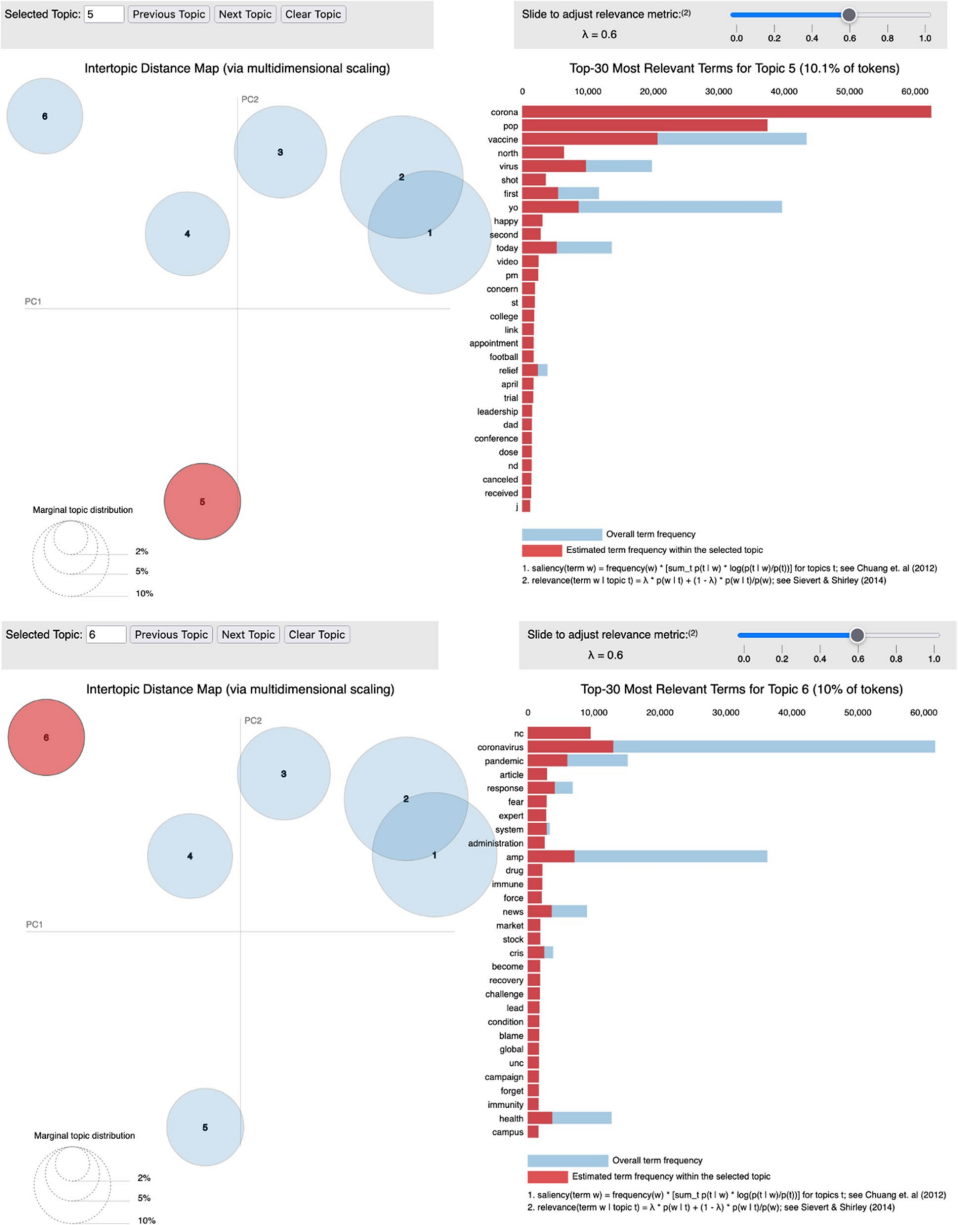
pyLDA visualization (Topic 5 & Topic 6). (pyLDA stands for Python Library for Latent Dirichlet Allocation Topic Model Interactive Visualization)

Four main topics were identified. Initially, six topics were selected based on coherence scores (see Fig. 4). Then, pyLDA visualization was used to finalize the topics and top terms. Following the recommendations from Sievert & Shirley [30], we set the relevance hyperparameter $\lambda$ at 0.6, and selected the topics and the top terms (see Table 2). Topic 5 and Topic 6 were dropped from the analysis due to low token size and difficulty for human interpretation (see Fig. 5). Based on the top words and the associated tweet content, the human analyst named and summarized each main topic (see Table 2). The top words from Topic 1 show that this topic was about getting the COVID vaccine and/or tests. Topic 2 was about politics and the Trump administration, the Presidential election, COVID relief bills, and China. Topic 3 was about COVID mitigation measures, such as face masks, social distancing, etc. Topic 4 featured a focus on COVID-related community/local issues, such as students, schools, and outbreaks. Accordingly, we named Topic 1 “COVID Vaccines,” Topic 2 “Politics,” Topic 3 “Mitigation Measures,” and Topic 4 “Community/Local Issues.”

**Table 2:** Top 20 words in the descending order of topics

Topic 1 “COVID vaccines”	Topic 2 “Politics”	Topic 3 “Mitigation measures”	Topic 4 “Community/local issues”
covid	trump	mask	case
get	coronavirus	covid	new
vaccine	american	home	coronavirus
like	china	flu	nc
im	death	year	vaccine
corona	people	stay	school
don’t	country	day	student
know	president	wear	testing
test	response	hand	north
virus	business	time	county
people	pandemic	corona	state
going	via	game	carolina
getting	amp	season	health
think	america	family	amp
really	white	amp	community
thing	million	old	update
tested	say	last	read
would	worker	one	today
take care	vote	wearing	patient
cant	government	party	rate

### Clustering tweets

**Fig. 6:**
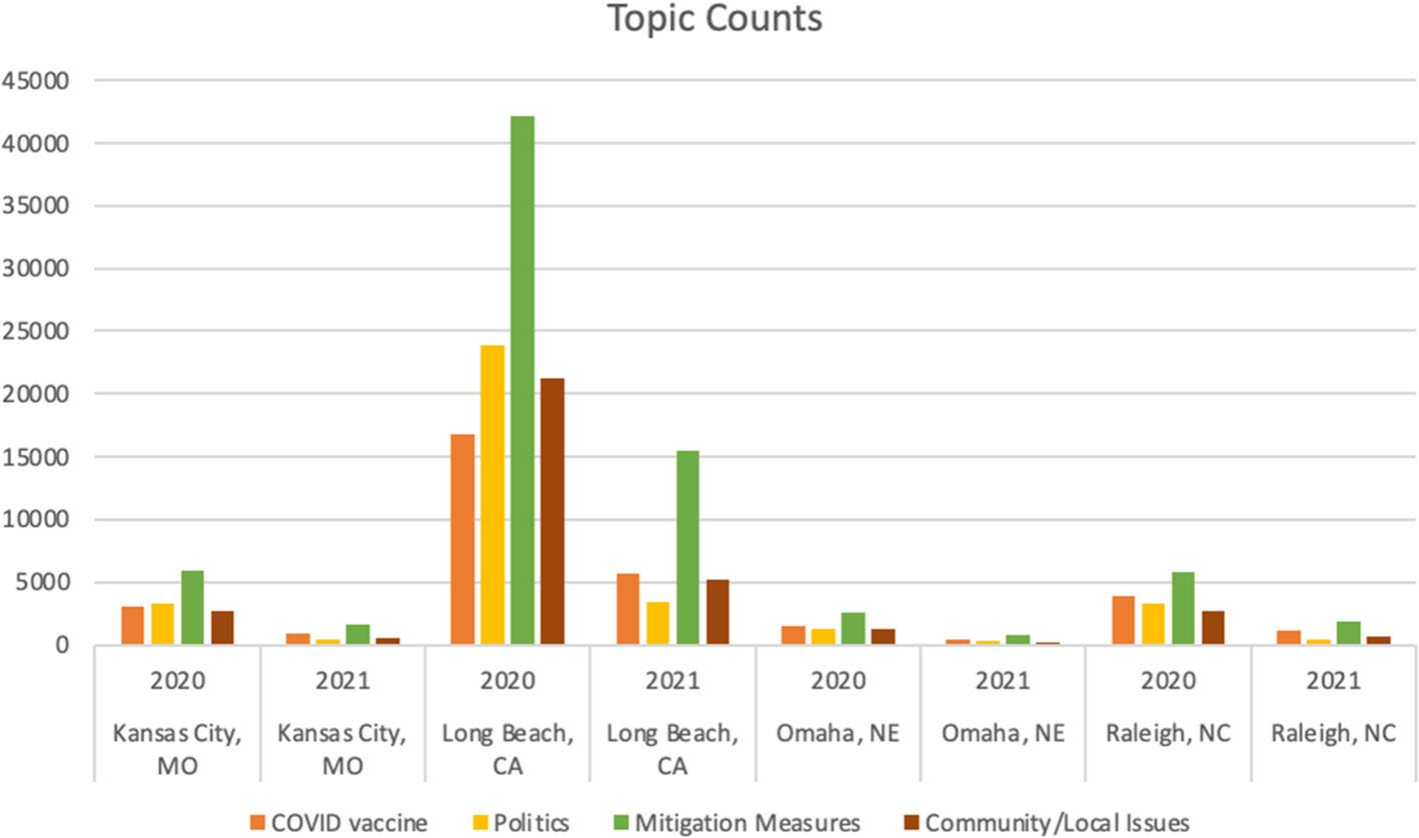
Tweet counts of topics

Based on output from the LDA + BERT clustering model, we visualized the topic distribution in each city in 2020 and 2021 (see Fig. 6). The counts are the numbers of tweets from each topic. Figure 6 shows that Long Beach had the most tweets. The general pattern of mitigation measures was discussed the most across all four cities in 2020 and 2021, followed by COVID vaccines. Long Beach in 2020 had slightly more tweets about community/local issues than COVID vaccines.

**Fig. 7:**
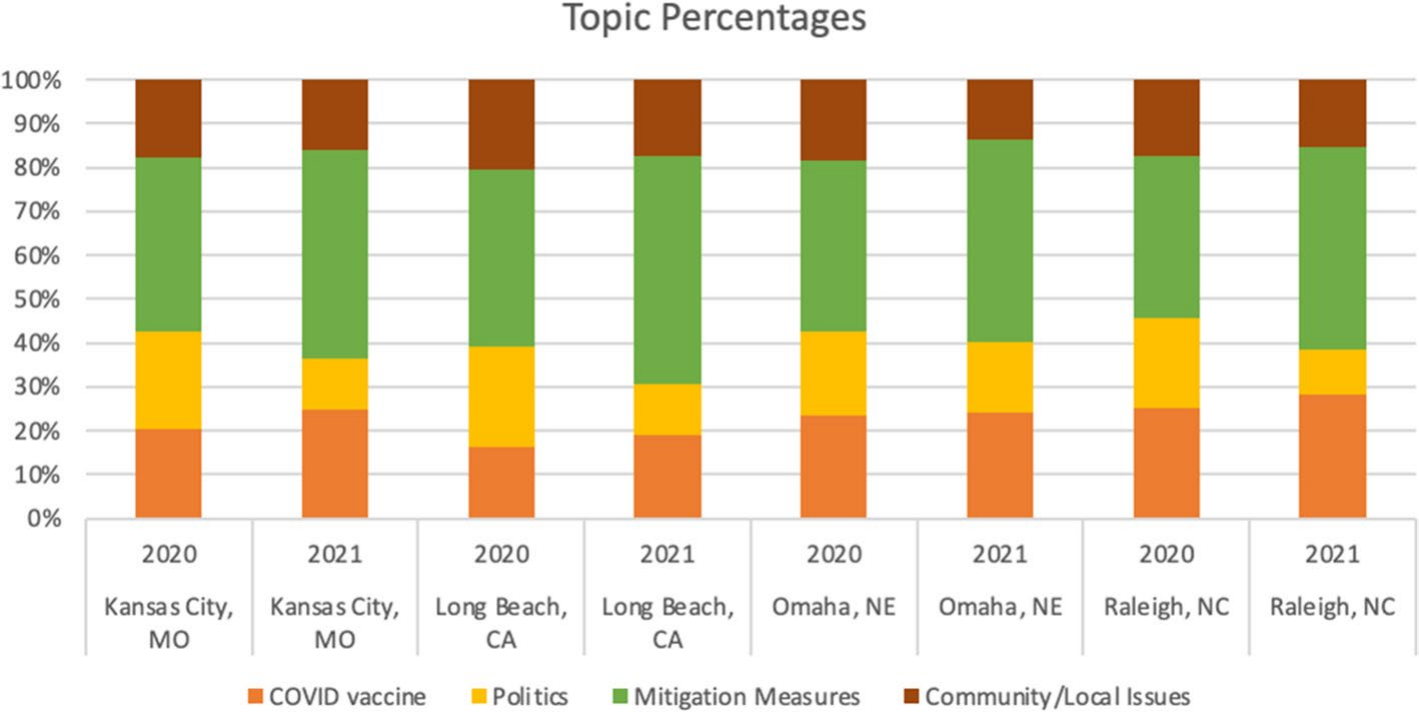
Tweet percentages of topics

The percentages reveal different patterns of topic distribution across the four cities in two years (see Fig. 7). While people generally tweeted less about COVID in 2021 than in 2020, there was an increased interest in mitigation measures and the COVID vaccine and less focus on political issues in 2021 than in 2020 among the tweets.

### Emotion analysis

**Fig. 8:**
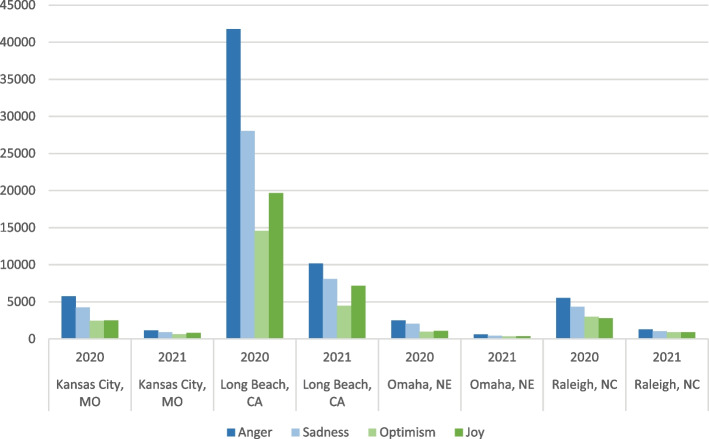
Counts of tweets of different emotions

**Fig. 9:**
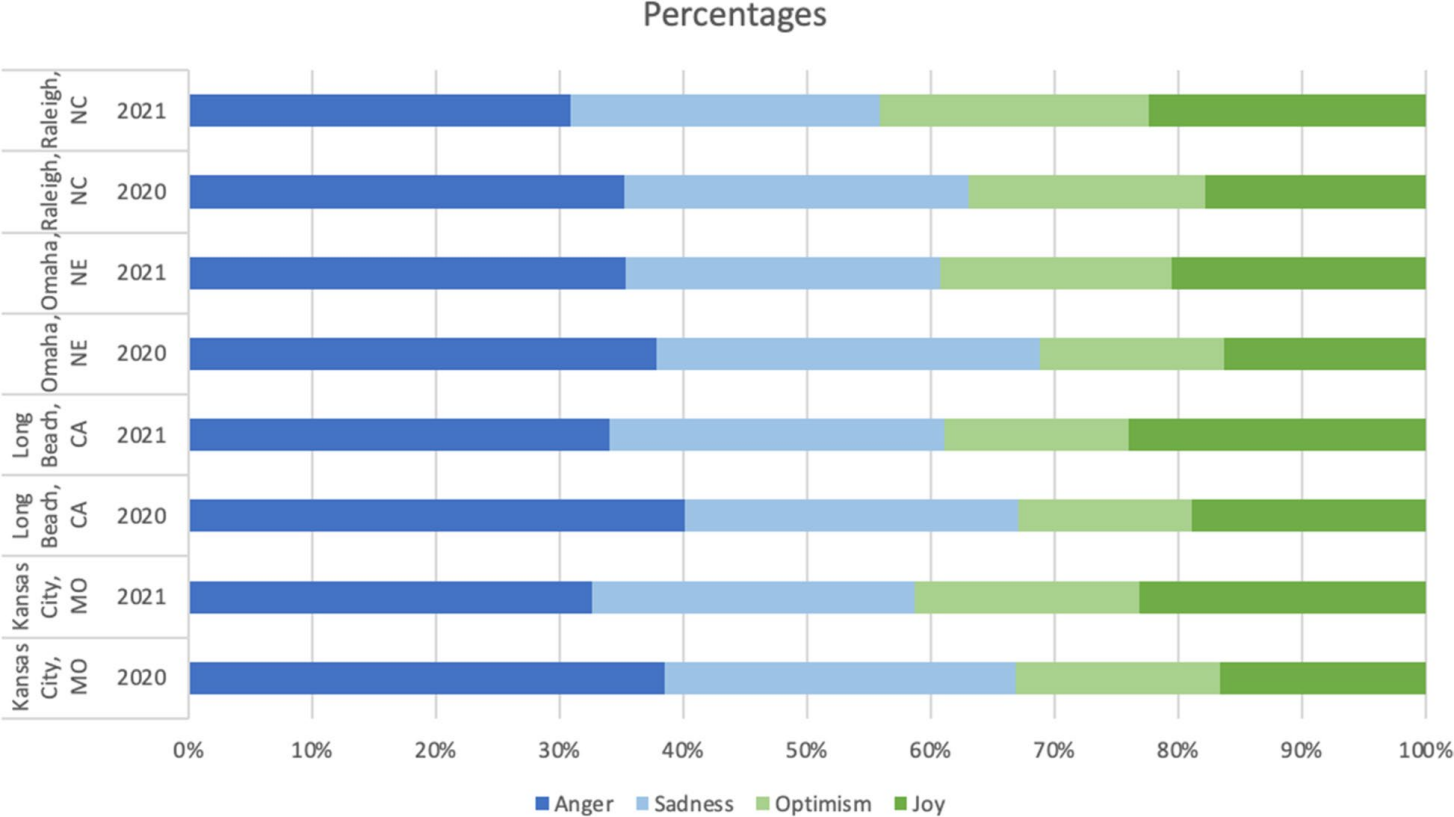
Percentages of tweets of different emotions

This section presents the output from the BERT emotional model. Figures 8 & 9 show that there were more tweets with negative emotion than positive emotion. People were more joyful and optimistic in 2021 than in 2020. The most positive city was Raleigh, NC. The most negative city was Omaha, NE, in 2020 and 2021. The angriest city was Long Beach, CA in 2020, and Omaha, NE, in 2021.

**Fig. 10:**
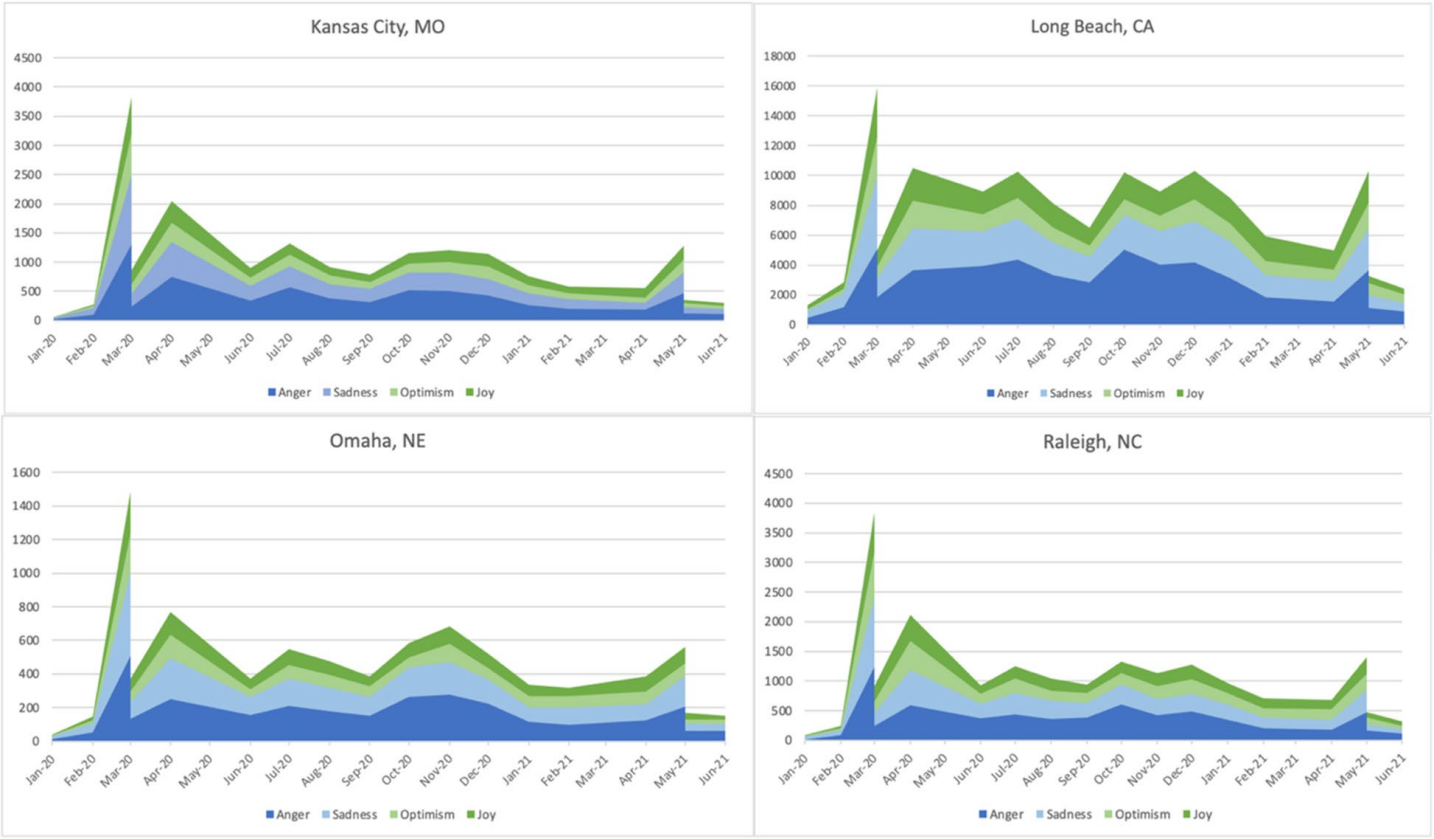
Month by month emotions counts 2020–2021

We also analyzed sentiment change over time in each city. Figures 10 show the change of tweet counts over time. February 2020, March 2020, September 2020, and May 2021 were the definitive moments in the sentiment distribution over time. All four cities had a sudden sharp increase in tweets about COVID in February 2020. This peak marked the beginning of the pandemic and featured a strong negative emotion. After a significant drop in COVID tweets in March 2020, there was an increase in April, which did not reach the peak amounts of tweets in March 2020. September 2020 marked a significantly larger drop in tweets in Long Beach, CA, than in other cities. May 2021 marked a significant increase in the tweets, coinciding with the rollout of the vaccine, and the number of tweets sharply fell in June 2021.

**Fig. 11:**
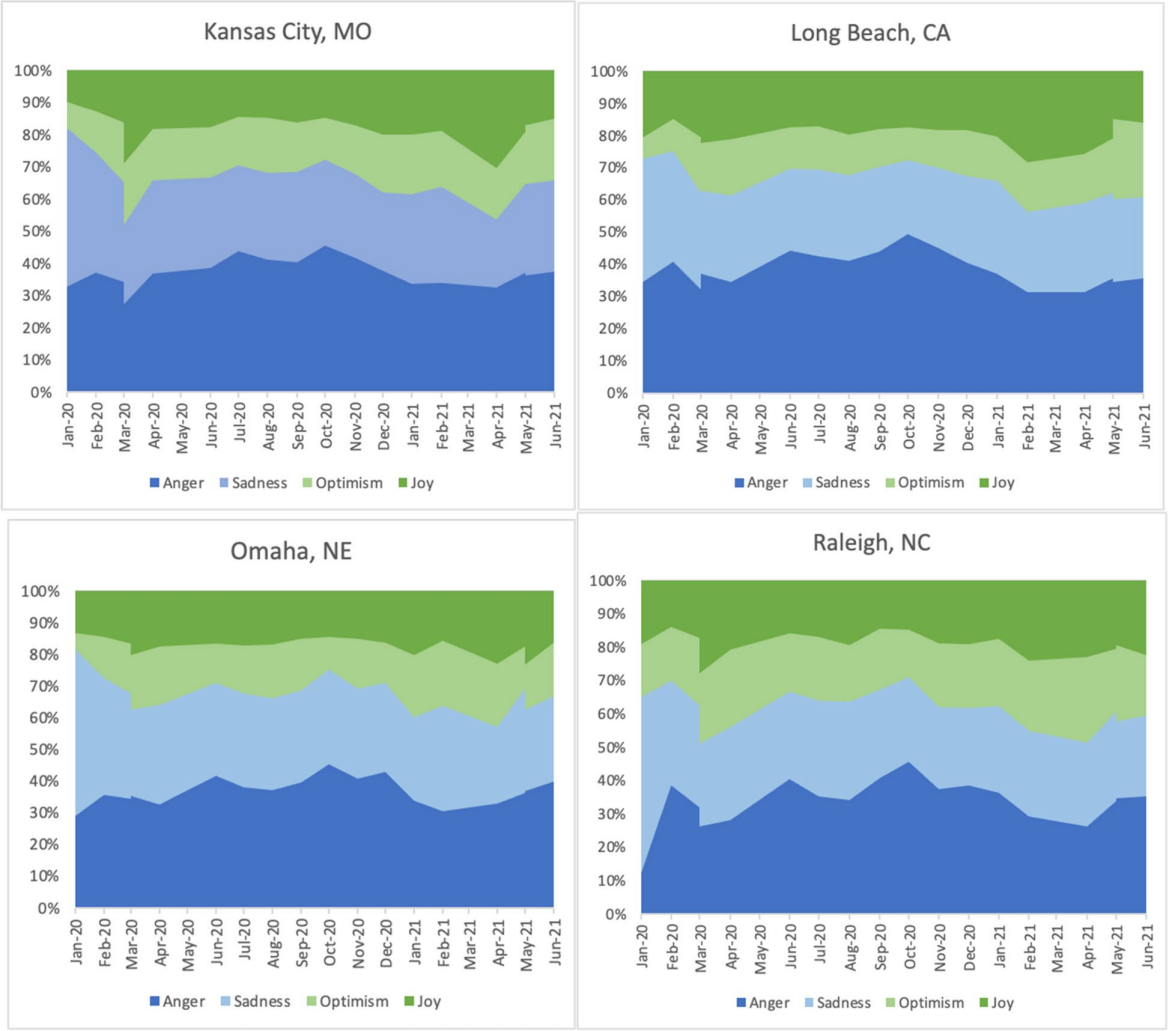
Month by month emotions percentages, 2020–2021

Figure 11 uses the percentages of emotion to indicate the change of moods among people who shared about COVID and vaccines on Twitter. In Kansas City, people suddenly became more joyful and optimistic in April 2021, when the COVID vaccine began to roll out. A similar surge of positivity in 2021 happened in February in Long Beach, CA, and April in Raleigh, NC, and Omaha, NE.

Combining the above two groups of charts, we can see that April and May in 2021 were the two critical months for the vaccination in Kansas City, MO, Omaha, NE, and Raleigh, NC. February and May 2021 were the two critical months in Long Beach, CA. There was a clear sign of optimism in the first month when vaccines were largely available to the public. However, this momentum was quickly replaced by an uproar of anger in May 2021. People’s emotions quickly changed from optimism and joy to anger just within the first month when people were allowed to get vaccines in Kansas City, MO, Omaha, NE, and Raleigh, NC. This change was more gradual and spread over three months in Long Beach, CA.

### Textual analysis

This section reports findings of the textual analysis that profiled each city.

#### Kansas City, MO:

Since the onset of the pandemic in the early months of 2020, users in Kansas City tweeted about their skepticism related to the existence and severity of COVID-19. Users questioned the legitimacy of rising COVID-19 numbers and the severity of symptoms, many comparing the virus to the seasonal flu. Users debated the necessity of the vaccine for those with healthy immune systems, especially since the long-term side effects of the vaccine are unknown. There was a misunderstanding about the purpose of the vaccine, many users believing that the vaccine could prevent contracting the virus altogether. While some users corrected this misnomer, many continued to stand by their choice not to be vaccinated and often used this point to rationalize their reasoning. Over time, users began to be vocal about the government's required lockdown and the negative effects on the economy and mental health. Users frequently debated the politics of COVID-19 and the vaccine rollout, acknowledging failures on the part of both the Trump and Biden Administrations, but also the varied messaging from government health agencies and pharmaceutical drug companies.

Despite rising COVID-19 numbers and increases in incidence in the Delta variant locally, many users longed for normalcy which meant a return to work and school and recreational activities, including professional sporting events. In addition, users in this area debated different therapeutic treatments for COVID-19 and the financial interests of both lawmakers and drug manufacturers. Ultimately, users expressed distrust of government and health organizations due to mixed messaging and inconsistent communication.

Here are a few examples (note: spelling or grammatical errors were not corrected; profanity was omitted):

“This covid bullshit is way out of hand and way over played.”

“78% of Covid patients hospitalized were obese. We don’t have a covid problem, we have a weight problem.”

“I am not getting a vaccine for a virus I already got and recovered from. Why would I get igg antibodies that I already have, injected into me along with who knows what with the potential of infertility side effects? Lol”

“Black people when you see white people coming with vaccines .. RUN. They didn’t rush to fix the water issue in Flint, Michigan did they? They can make a vaccine but can’t cut a monthly check to help it’s citizens impacted by this virus?”

“ … , I’m glad she go her vaccine. My problem is with the government’s poor rollout of it. I have family members older than her and with health issues who still don’t have it. I’m allowed to be frustrated with the system.”

“Highly contagious Delta variant detected in Kansas City wastewater samples. … ”

#### Long beach, California:

In this urban area, many users began the pandemic by sharing fears of contracting COVID-19 and experiencing long-term effects or death from the virus. Some users had a fear of contracting COVID-19 and/or spreading it to their loved ones. Users discussed the severity of the virus symptoms, and some compared the virus to the flu. There was little discussion or debate related to the existence of the virus; instead, users debated the safety precautions instituted by the state government, like mask mandates or economic lockdowns. These types of discussions often turned to the politics of COVID-19 and the evaluation of administration efforts to contain the pandemic in the United States. Users also discussed where the virus originated, both in the world and in California. Users in Long Beach specifically debated President Trump’s handling of the virus and the development of the COVID-19 vaccine.

As the pandemic progressed, users discussed the impact of COVID-19 on the economy and the stock market. California experienced a very strict statewide lockdown, especially in comparison to other states; due to the strict guidelines, many users raised concern about the future of California’s economy and debated how the state would “bounce back.” In response to rising COVID-19 numbers both in the state and the nation, users compared the virus to gun violence and the need for reform. Users reported updates of local COVID-19 outbreaks and county safety protocols that might affect others in the area. Many users debated how COVID-19 is transmitted, especially after being vaccinated. This led users to debate how COVID-19 numbers are reported, and many complained about the lack of consistency or that there was no standard for reporting.

Here are a few examples (note: spelling or grammatical errors were not corrected; profanity was omitted):

“Lockdown open up, don’t wear mask must wear mask, there won’t be a vaccine we must wait for a vaccine.. Wth is all have to say. Last week this guy was saying we must stay locked down. Now he’s saying open up. And ppl wonder why we question the experts.”

“Or is it because this is flu season, and since covid is similar to the flue, there are a lot of possible false positive test? Is it possible that if you have the flu, you can still test positive for Covid?...”

“Was Trump right when he ran around the country in January saying it would be over after election day? When we had deaths pile up to 600k after. Was he right when he told people to take hydrochlorquine and then passed on it when he got covid? He mishandled this thing badly.”

“Covid numbers are going up nationwide even here in Orange County. … ”

“Again, you’re just flat wrong. CA has one of the slowest spreads. Like New York, it was hit hard early but has been improving. And prior to the pandemic, CA was in excellent financial shape for the 1st time in a long time.”

“AP in reporting COVID cases in different countries, wouldn’t it be more informative to report the per capita rates instead of, or at least in addition to, the absolute rates?”

#### Omaha, Nebraska:

This urban area began the pandemic with a lot of doubts and hesitation related to the severity of COVID-19. Many users discussed the severity of the virus in relation to the flu, and some believed that the pandemic was political propaganda. Users in this region were especially upset that the pandemic interrupted national sporting events. Many attributed “getting back to normal” to being vaccinated and encouraged others to choose the vaccine when it was their turn. However, there were many users who shared vaccine hesitancy due to misinformation. Hesitancy then led to political debate and who is to blame for the pandemic. Interesting in this localized data is the attention on the inconsistent public health messaging related to masks, social distancing, and the vaccine. While many pointed to the media, social media was acknowledged as a major problem in spreading misinformation.

While the necessity of economic lockdown was debated among these users, many looked ahead and wondered what was next for their region. Users argued whether another wave of COVID-19 would hit and if there would be mutations of the virus. Fear of another wave led users to discuss vaccine mandates and whether Americans should have the freedom of choice during this public health crisis. In addition, vaccine mandates and emergency approval by the FDA fueled users’ lack of confidence and trust in government agencies and pharmaceutical companies, questioning why they wouldn’t have the right to choose.

Here are a few examples (note: spelling or grammatical errors were not corrected; profanity was omitted):

“Daily reminder that influenza kills 40-50,000 people per year WITH a vaccine.”

“How the hell can all deaths in 2020 equal the same amount of deaths (10 years prior) but al from covid in 2020?.. Did all other illnesses, accidents, heart attacks, old age and etc. Just not happen? .. Wake Up People!! .. Or are you that Gullible!!..??…”

“Well we won’t be playing this weekend. Tournament canceled. Covid is too out of control. I am disappointed, but it’s the right decision. I’m guessing they will probably be pulling youth sports (except HS) on Monday. People here can’t stay out of the bar and just wear a mask.”

“I think the point here is that Joe isn’t consistent in his actions. Also, the CDC says to have your mask on indoors regardless of vaccine status. PLUS … The vaccine doesn’t stop the spread, it only lessens the symptoms.”

“@GovRicketts please do something about this overbearing Omaha City Council and the continuous undermining of our rulings and decisions on Covid-19. They have once again extended a mandatory mask mandate city wide and it needs to stop and stop now.”

#### Raleigh, North Carolina:

To begin the pandemic in this part of the country meant that many users had questions about the severity of COVID-19 and how it differed from the flu. As more state governors chose to close down local economies, users had plenty to say about it. Users debated the economic effects of the national lockdown on the local economy and the necessity of safety precautions when numbers were relatively low. As the potential for a vaccine became a reality, users debated whether or not the vaccine would be effective and in what capacity. Those who chose not to get the COVID-19 vaccine often pointed out that they had been vaccinated, e.g., MMR, tetanus, and were (usually) in favor of vaccines, but in this case were opting out due to the “warp speed” of the development and production of the vaccine and the lack of proper scientific trials to guarantee its safety.

Often, any discussion of COVID-19 turned to politics. In the case of Raleigh, users debated the handling of the virus and the vaccine rollout by both President Trump and President Biden. Users in this area also discussed immigration and whether immigrants contributed to the United States’ pandemic numbers and deaths. Many users discussed the increasing violence against Asians and the dangerous rhetoric used by political leaders. Users seemed to be unhappy with their local government officials and the decisions being made related to COVID-19. The local economy was a popular topic of conversation; many users shared concern over local businesses and their ability to rebound after the lockdown. Users discussed different COVID-19 therapeutic treatments and the effectiveness of safety precautions in the midst of rising numbers nationally. Finally, many debated the potential of a vaccine mandate and whether or not vaccine passports should be used.

Here are a few examples (note: spelling or grammatical errors were not corrected; profanity was omitted):

“It’s wild how much covid has impacted my mental health. Gotta get out of the house.”

“You ask why I’m not going to get the vaccine that was supposed to take at least a yr or two down there road to complete, yet it is approved 8 mths later when they are still struggling w/ the flue&still not 100 on it!? Yeah no thank you.”

“Analysts out there acting surprised the vaccine got approved. The US govt doesn’t give a fuck about Americans living in poverty, jobless and hungry. You think they care if they inject millions with some vaccine that’ll fuck you up worse than covid does?”

“Trump’s admin rolling out a vaccine and half of his supporters not taking it is a very clear policy and messaging failure.”

“While simultaneously allowing thousands of Covid positive illegal immigrants into our country.”

“I need them to mandate this vaccine so we can get this new world order on schedule.”

“North Carolina has handled COVID better than New Jersey and our economy will benefit as business and people move to the Triangle.”

## Discussion

Public health crisis management is challenging because health is such a personal topic, often influenced by family and culture. As healthcare continues to move to patient-centered practices [39, 40], and patients increasingly utilize the Internet [41, 42], it stands to reason that public health communicators should engage in social listening and glean insights from target populations to better engage with them. Engaging in social listening allows practitioners to track public opinion and predict trends in consumers’ attitudes and behaviors. It is important to invest in such information so that health communication can be data-driven and become most effective.

This research project sought to examine Twitter posts from mid-size cities across the United States with high percentages of African Americans to gather insight into the COVID-19 vaccine and related hesitancy. The presented data analysis process examined both the breadth and depth of data. The NLP analysis discovered macro topic and emotional trends, and the human textual analysis, assisted and informed by NLP analytics, created market “profiles” for the cities. The following discussion offers strategic recommendations based on these findings.

Findings from this study suggest public health agencies should seek to engage the public by implementing three strategies. First, the objective of communicating science is to give people a sense of control and empower them to become capable co-producers of knowledge and make good health decisions. The Twitter data from Kansas City revealed that this geographical area specifically does not understand the science related to COVID-19, including the severity of the virus, how the virus is contracted and/or spread. Public health messaging should focus on how COVID-19 differs from the flu and the potential of long-term health effects. In Raleigh, North Carolina, it should be said that many who were refusing the COVID-19 were not “anti-vaxxers” but instead were “refusers.” This is an important difference to note as this population was targeted for persuasive messaging. Refusing the COVID-19 did not then make one against vaccines, some users argued. Instead, users weighed the risks of contracting the virus versus the unknown long-term side effects of the vaccine, which often determined their choice to be vaccinated. Thus, the public health agencies need to listen to the concerns, and purposefully communicate the science around vaccines.

Second, health communication should seek to build trust locally and among local leaders on health issues that affect community residents and have a strong local theme and local relevance. One of the main topics discovered was the importance of community and users’ investment in local issues. Specifically, the Twitter data from Omaha revealed that this geographical area valued its independence and did not like government interference. To minimize local resentment to national pushes, appealing to “hometown values” is a gesture of “expressed empathy,” i.e., recognition of stakeholders’ needs. For example, the local Twitter data showed that users were very community-oriented and especially concerned about the future of the local community. Thus, appealing to rebuilding the local communities and putting in the sense of pride in the messaging may help strike a more positive tone and counter the negativity growing from economic and mental health concerns.

Third, timing is important to health communication. The emotion analysis suggested that vaccination efforts in these cities missed the opportunity of capitalizing on people’s excitement in early 2021. During these earlier days of vaccination rollout, local public health agencies might not have adequately recognized and accommodated difficulties that discouraged and limited these communities from accessing vaccines. Perhaps justifiably, users who faced challenges to getting the vaccine were angry. The data in June 2021 clearly shows a drop of people’s interest in discussing the vaccine and persistent anger. Arguably, this change of emotion availed opportunities to vaccine hesitancy and disinformation, which disproportionally affected people of color.

### Limitation

Findings from this study may be limited by the sampling bias of the data. This is a common problem to sampling from the social media. Twitter users are not necessarily representative of the public or all local communities in a city. According to a Pew Research Center’s report, U.S. adult Twitter users are younger, more educated, wealthier, and more likely to be Democrats than the general population [27]. On average, 10% of U.S. users generate 80% of the tweets [27]. Although the geo-location function was applied to scrape many tweets in hopes of capturing a diverse range of demographics, the sampling procedure did not ensure the tweets were originated by race minorities. Thus, any generalization of the results should be cautioned.

## Conclusions

Public health agencies inevitably compete for users’ attention [43] in a decentralized information environment. So often, they are ill-prepared to communicate with target audiences clearly and effectively. Local community stakeholders have their own concerns and unique culture that must be included in public health communication so public health agencies can be perceived as credible and trustworthy. Listening to consumers is a common practice in many industries, including marketing and advertising [44, 45], real estate [46], travel [47, 48]. Moreover, health communication should be more regular since science and medicine are complex topics that laypeople (most stakeholders) do not understand. Due to low health literacy [49, 50], it is not enough to communicate with local communities when there is an emergency like COVID-19 or the introduction of a vaccine. Instead, public health entities must regularly listen to and communicate with target audiences.

However, one of the obstacles is scalability: it is almost impossible for manual analyses to “listen to” constant streams of community feedback, especially during public health crises. To address this challenge, the current study presents a method of combining the strength of machine learning and human interpretation. The presented case study demonstrated that this method could efficiently reduce a large amount of community feedback (e.g., tweets) by NLP and ensure contextualization and richness thanks to human interpretation.

## Supplementary Information

**Additional file 1:** **Appendix.** Extracting Themes and Topic Terms.

## Data Availability

The Twitter data used in the paper is publicly available via Twitter. Readers can contact wanye@umsystem.edu to access data used in this study.

## Abbreviations

AI: Artificial intelligence
API: Application programming interface
BERT: Bidirectional encoder representations from transformers
BoW: Bag-of-words
DL: Deep learning
LDA: Latent dirichlet allocation
ML: Machine learning
NLP: Natural language processing
NPMI: Normalized point-wise mutual information
pyLDA: Python library for latent dirichlet allocation topic model interactive visualization
TF-IDF: Term frequency-inverse document frequency
Word2Vec: Word to vector
