# Supplementary material for: A case study of using natural language processing to extract consumer insights from tweets in American cities for public health crises
Source: BMC Public Health. 2023 May 24;23:935. doi: 10.1186/s12889-023-15882-7 (PMC10206352; doi:10.1186/s12889-023-15882-7)
Supplement: Supplementary file 1 — Additional file 1: Appendix. Extracting Themes and Topic Terms. [file 12889_2023_15882_MOESM1_ESM.docx]

Appendix.

*Extracting Themes and Topic Terms*

A document is a tweet, and a tweet consists of words. Topics are latent semantic spaces characterized by a distribution of words, and a tweet has a mixture of latent topics. Given an input corpus D (d $\in$ D) with *V*unique words from *M* documents, each document d contains a sequence of n words d = {W1, W2, …, W_N_}, n $\in${1, 2, …, N}. Given a topic number k, k $\in${1, 2, …, K}, the generative process will create documents based upon per-document topic distribution and per-topic word distribution. We used coherent analysis to determine k, and the final model had four topics.

α is the per-document topic distribution; it indicates the likelihood that a document contains topic Zk, k $\in$ {1, 2, …, K}. β is the per-topic word distribution. θd is a multinomial distribution of documents drawn from a Dirichlet distribution with the parameter α. ϕ_k_, k $\in$ {1, 2, …, K} is a multinomial distribution of words in a topic drawn from a Dirichlet distribution with the parameter β. For each word position n $\in$ {1, 2, …, K} in a document, select a hidden topic Z_n_ from the multinomial distribution parameterized by θ_d_. And then select W_n_ from ϕ_Zn_.
